# Glycogenic Hepatopathy: A Case Report of a Rare Complication in Uncontrolled Diabetes

**DOI:** 10.1002/ccr3.71079

**Published:** 2025-10-01

**Authors:** Mais Musleh, Amani AlMokbel

**Affiliations:** ^1^ Department of Hematology, Faculty of Medicine Al Assad University Hospital Damascus Syria; ^2^ Department of Hematology, Faculty of Medicine Al‐Mouwassat University Hospital Damascus Syria

**Keywords:** diabetes, glycogenic hepatopathy, liver biopsy, rare

## Abstract

Glycogenic hepatopathy (GH) is an underrecognized yet reversible cause of hepatomegaly and transaminase elevation in adolescents with poorly controlled type 1 diabetes mellitus (T1DM). While liver biopsy is often utilized to exclude other hepatic pathologies, it can also aid in confirming GH when noninvasive findings are inconclusive. We present a case of an 18‐year‐old male with type 1 diabetes, whose poorly controlled condition led to the development of GH. The diagnosis was confirmed through a liver biopsy, emphasizing the significance of early detection and proper glycemic management in preventing irreversible liver damage.

**Abbreviations**
GH: Glycogenic hepatopathy
H&E stain: hematoxylin and eosin stain
PAS stain: Periodic acid‐Schiff stain
T1DM: Type 1 Diabetes Mellitus
T2DM: Type 2 Diabetes Mellitus

## 1 Introduction

Glycogenic hepatopathy (GH) is a rare and possibly underdiagnosed complication most commonly observed in children and young adults with poorly controlled Type 1 Diabetes Mellitus (T1DM). However, it can also occur in individuals with Type 2 Diabetes Mellitus (T2DM) [1, 2]. GH is characterized by the reversible accumulation of excess glycogen within hepatocytes, resulting in hepatomegaly and transient elevations in liver enzymes, particularly transaminases. The diagnosis of GH is confirmed through liver biopsy with glycogen staining, typically using hematoxylin and eosin (H&E). GH is a component of Mauriac syndrome (MS), a condition marked by growth retardation, delayed puberty, a cushingoid appearance, hepatomegaly with abnormal liver function tests, and hypercholesterolemia [2, 3]. In this report, we present a case of an 18‐year‐old male with hepatic glycogenosis associated with poorly controlled diabetes, highlighting the importance of liver biopsy in diagnosing GH.

## 2 Case History

An 18‐year‐old male was diagnosed with T1DM at age three and has been managing it with insulin, exhibiting consistently poor glycemic control due to inadequate therapeutic adherence. During his adolescence, he had multiple hospital admissions for diabetic ketoacidosis, with persistently elevated glycated hemoglobin levels above 10%. He was subsequently referred to the diabetes department for optimized management.

At the time of referral, the patient was receiving multiple daily insulin injections, comprising a basal–bolus regimen with a total daily dose of approximately 1 IU/kg. Additionally, he intermittently administered pre‐meal mixed insulin in an unscheduled and erratic manner. Despite these interventions, glycemic control remained suboptimal.

## 3 Methods

Upon admission, the patient had a height of 134.5 cm and a weight of 36 kg (< 3rd percentile for age and sex), with a body mass index (BMI) of 18.9. He presented with a cushingoid facial appearance and showed no signs of secondary sexual characteristics, indicating that he was at Tanner stage 1. A physical examination revealed hepatomegaly, with the liver palpable 4 cm below the subcostal margin. There were no signs of jaundice, splenomegaly, peripheral edema, or ascites.

Initial laboratory evaluations indicated a serum glucose level of 238 mg/dL, glycated hemoglobin of 114 mmol/mol, total cholesterol of 310 mg/dL, and triglycerides of 274 mg/dL. The acid–base balance was normal, with a pH of 7.39 and serum bicarbonate at 24 mmol/L. Liver function tests showed significantly elevated transaminases—alanine aminotransferase (ALT) at 522 U/L and aspartate aminotransferase (AST) at 4040 U/L—while alkaline phosphatase and total bilirubin remained within normal limits. Gamma‐glutamyl transferase (GGT) was also elevated at 87 U/L. Prothrombin time was within the normal range. Serum lactate was notably elevated at 46 mg/dL (normal: 4.5–20 mg/dL), and this elevation persisted beyond the timeframe typically seen in diabetic ketoacidosis, further supporting the diagnosis of glycogenic hepatopathy (GH). Serum ammonia was elevated at 83 μmol/L (normal < 50 μmol/L), without clinical signs of hepatic encephalopathy, and was attributed to metabolic dysregulation associated with hepatic glycogen overload.

Further investigations ruled out autoimmune hepatitis (negative autoantibodies) and infectious hepatitis (negative serologies for Epstein–Barr virus, cytomegalovirus, hepatitis A, B, and C viruses, and HIV). Hemochromatosis was excluded by normal iron studies, and Wilson's disease was ruled out by normal ceruloplasmin and copper levels.

The patient reported a history of generalized abdominal pain, abdominal distension, and intermittent watery diarrhea, unrelated to food intake. His medical history was significant for poorly controlled diabetes mellitus. He denied alcohol, tobacco, or illicit drug use. Laboratory evaluation for diarrhea included negative microbiological studies of feces and normal levels of serotonin, chromogranin A, gastrin, vasoactive intestinal peptide, glucagon, and urinary 5‐hydroxyindoleacetic acid.

Ultrasonography confirmed significant hepatomegaly. A CT enterography revealed no structural abnormalities in the small intestine. Upper gastrointestinal endoscopy, ileocolonoscopy, and histopathological examination of duodenal, colonic, and ileal biopsies were unremarkable.

**FIGURE 1:**
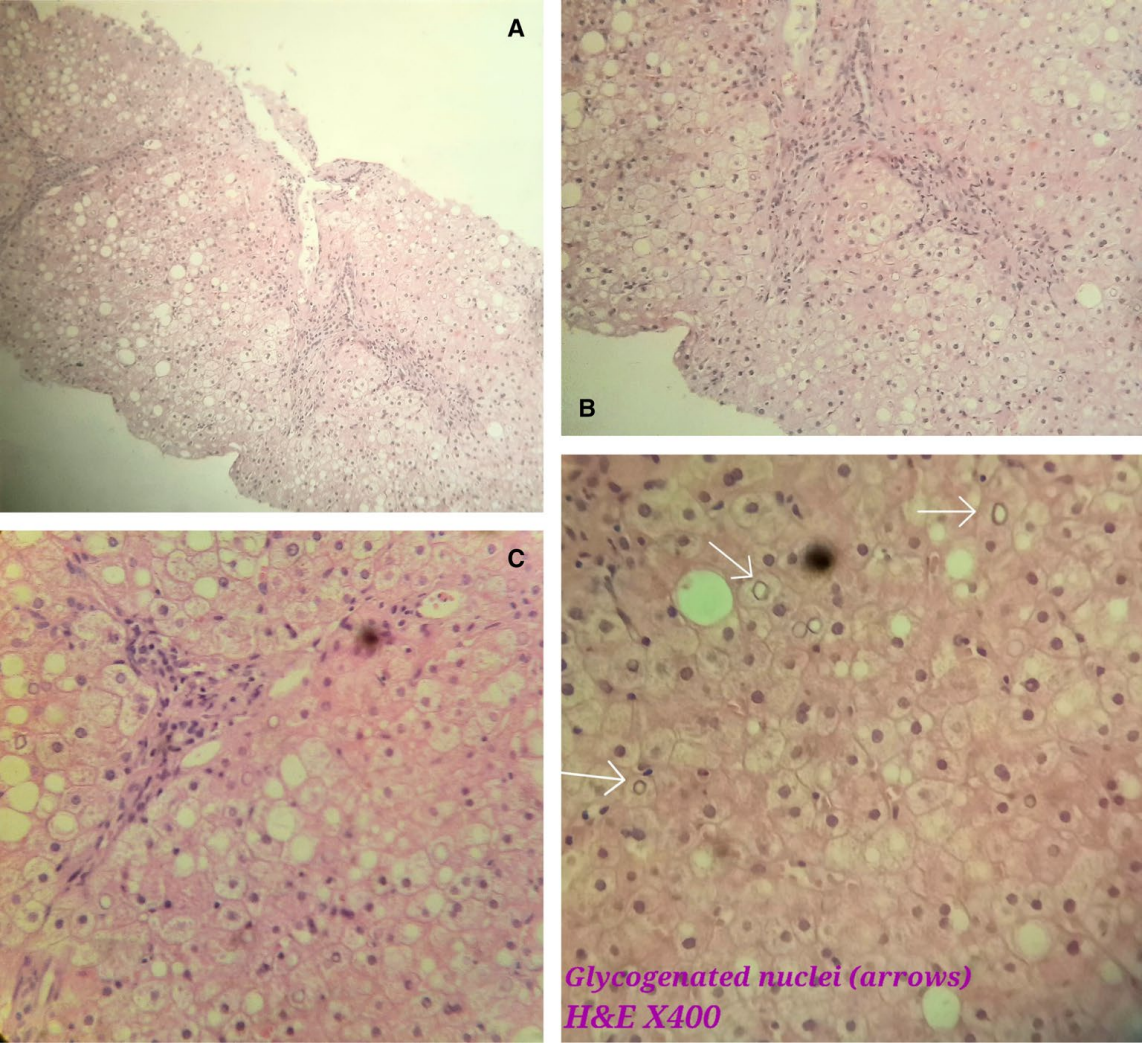
(A–C) and glecogen nuclei: Liver biopsy showed mild distortion of liver architecture. The hepatocytes exhibited diffuse swelling and enlargement, characterized by intracytoplasmic vesicles, centrally located nuclei (arrow), and mild steatosis, arranged in a mosaic pattern. Fibrosis was noted within the portal spaces. (A) H&E stain, magnification ×100. (B, C) Glecogen nuclei: H&E Stain, magnification ×400.

**FIGURE 2:**
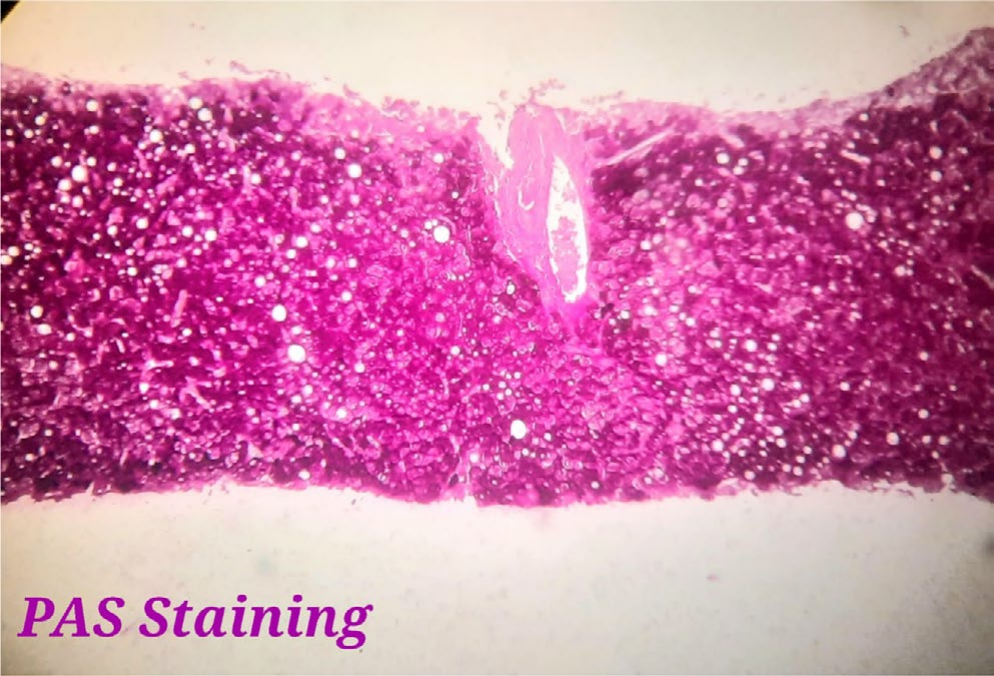
Liver biopsy showed Intracytoplasmic glycogen which disappears after digestion with diastase. PAS stain, magnification ×400.

**FIGURE 3:**
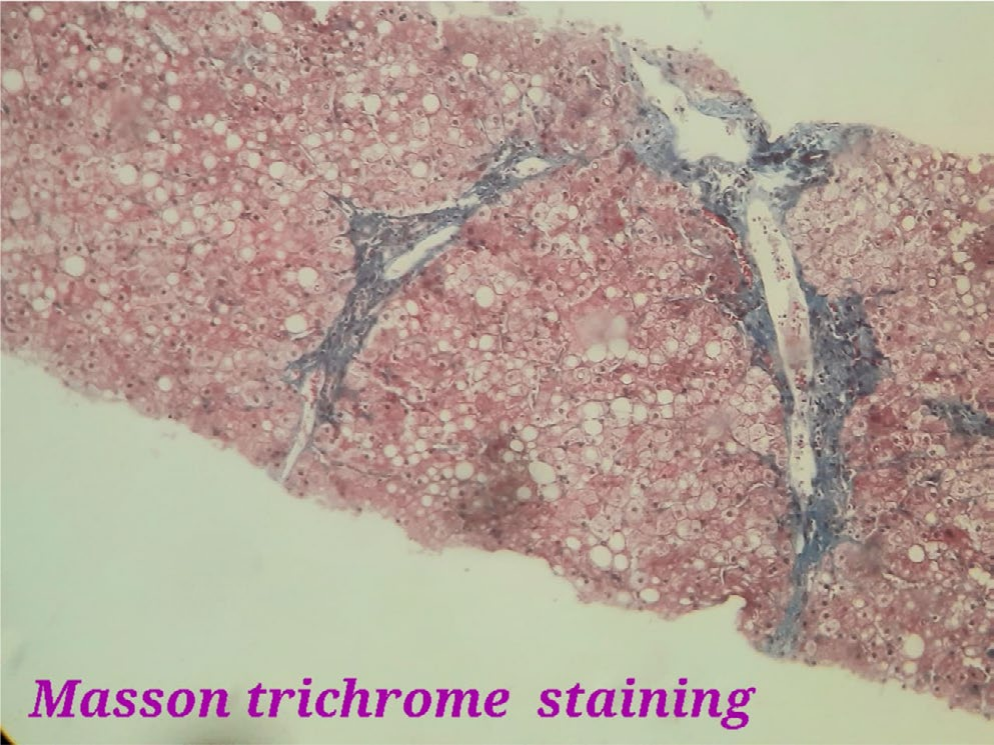
Liver biopsy showed portal–portal fibrosis. Trichrome Masson staining, magnification ×400.

A liver biopsy was conducted to assess the hepatic parenchyma. Optical microscopy demonstrated mild distortion of liver architecture. The hepatocytes exhibited diffuse swelling and enlargement, characterized by intracytoplasmic vesicles, centrally located nuclei, and mild steatosis, arranged in a mosaic pattern. Fibrosis was noted within the portal spaces, and these findings were confirmed using Hematoxylin and Eosin (H&E) staining, as illustrated in Figure 1. Periodic acid‐Schiff (PAS) staining revealed significant intracytoplasmic glycogen accumulation, which disappeared following diastase digestion, as shown in Figure 2. Furthermore, Trichrome Masson staining confirmed the presence of portal‐portal fibrosis, as shown in Figure 3.

## 4 Conclusion and Results

The diagnosis of glycogenic hepatopathy (GH) was established within the framework of Mauriac syndrome (MS). Management centered on optimizing glycemic control and reinforcing strict adherence to an appropriate dietary regimen. The patient's insulin dosage was progressively increased to 2.3 IU/kg/day to improve metabolic control. Additionally, symptomatic management included the initiation of octreotide and loperamide to address persistent diarrhea.

During follow‐up, the patient demonstrated a decreased frequency of diarrhea and showed gradual improvements in glycemic control.

## 5 Discussion

Glycogenic hepatopathy (GH) is characterized by the reversible accumulation of excess glycogen in hepatocytes, leading to hepatomegaly and transient elevations in liver enzymes, particularly transaminases. While GH is a well‐recognized condition that can occur at any age, it may present without the full spectrum of features associated with Mauriac syndrome. Despite numerous case reports and several series, the exact prevalence of GH remains unknown. However, it is considered the leading cause of hepatomegaly in children and adolescents with T1DM [4, 5]. GH has been referred to by various terms, including hepatic glycogenosis, liver glycogen storage, and diabetes mellitus‐associated glycogen storage hepatomegaly [6, 7, 8, 9, 10]. In this case, we diagnosed an 18‐year‐old male with poorly controlled T1DM and GH.

The pathogenesis of glycogenic hepatopathy (GH) is not yet fully understood but is largely driven by marked fluctuations in plasma glucose levels, characterized by alternating episodes of hyperglycemia and hyperinsulinemia [1, 2, 4]. Elevated glucose enhances hepatocellular uptake, while hyperinsulinemia activates glycogen synthase, promoting the conversion of glucose‐6‐phosphate into glycogen. Additionally, in poorly controlled type 1 diabetes mellitus (T1DM), increased serum cortisol—often a counter‐regulatory response to hypoglycemia—may further augment glycogen accumulation. This complex interplay of glucose and hormonal regulation is central to GH development, though it remains unclear why only some patients develop the condition [4, 11].

The diagnosis is confirmed through liver biopsy and glycogen staining, using H&E stain. Histologically, the hepatocytes exhibit marked glycogen accumulation, appearing diffusely swollen with pale cytoplasm and prominent cell membranes. These changes are frequently accompanied by nuclear displacement to the cell periphery, sinusoidal compression, glycogenated nuclei, and the presence of giant mitochondria. PAS staining distinctly highlights the glycogen accumulation, which is absent following diastase digestion. Typically, the liver biopsy reveals minimal fat content, limited inflammation, sparse or absent spotty lobular necrosis, preserved liver architecture, and minimal fibrosis [6, 7, 8].

In this case, GH was diagnosed through liver biopsy, which revealed several defining histological features, as depicted in the figures. Optical microscopy showed mild distortion of liver architecture. The hepatocytes displayed diffuse swelling and enlargement, characterized by intracytoplasmic vesicles, centrally located nuclei, and mild steatosis, arranged in a mosaic pattern. Fibrosis was observed within the portal spaces, with confirmation provided by H&E staining. PAS staining highlighted significant intracytoplasmic glycogen accumulation, which was eliminated after diastase digestion. Additionally, Trichrome Masson staining confirmed the presence of portal‐portal fibrosis.

Several studies have highlighted the rarity and diagnostic challenges of glycogenic hepatopathy (GH). Although GH is traditionally considered reversible with improved glycemic control, the presence of fibrosis in our patient raises important clinical concerns [12]. Current literature suggests that while liver enzyme abnormalities and glycogen accumulation often resolve rapidly with tighter glycemic control, fibrosis may persist, representing a potentially irreversible complication that warrants further investigation [13, 14, 15]. This distinction underscores the need for early diagnosis and prompt intervention.

In our case, the imperative of maintaining strict glycemic control to prevent complications like GH was evident. The patient was counseled on the critical importance of adhering to diabetes management protocols and was initiated on octreotide and loperamide therapy for symptom management.

In conclusion, glycogenic hepatopathy (GH) is a notable yet potentially reversible complication of poorly controlled type 1 diabetes mellitus (T1DM). Successful management depends on early recognition and prompt intervention, underscoring the critical role of strict glycemic control and appropriate dietary measures. While liver biopsy is primarily utilized to exclude alternative causes of hepatomegaly and liver dysfunction, GH should remain an important consideration in the differential diagnosis. GH may lead to serious complications such as fibrosis, steatosis with cardiovascular implications, and significant pubertal delay and growth restriction. Ongoing research is needed to better understand the pathogenesis and long‐term outcomes of GH, as well as to inform the development of more effective therapeutic approaches. This further highlights the necessity of comprehensive patient education and diligent adherence to diabetes management protocols to minimize the risk and burden of GH.

## Author Contributions

**Mais Musleh:** conceptualization, data curation, formal analysis, project administration, resources, software, visualization, writing – original draft, writing – review and editing. **Amani AlMokbel:** data curation, resources, supervision.

## Disclosure

Guarantor: Amani AlMokbel is the guarantor of this work.

## Ethics Statement

The authors have nothing to report.

## Consent

Written informed consent was obtained from the patient for publishing this case report and any accompanying images. A copy of the written consent is available for review by the Editor‐in‐Chief of this journal on request.

## Conflicts of Interest

The authors declare no conflicts of interest.

## Data Availability

The authors have nothing to report.
